# Displacement of Pathogens by an Engineered Bacterium Is a Multifactorial Process That Depends on Attachment Competition and Interspecific Antagonism

**DOI:** 10.1128/IAI.00020-16

**Published:** 2016-05-24

**Authors:** Fitua Al-Saedi, Daniel Henry Stones, Diana Pereira Vaz, Anne Marie Krachler

**Affiliations:** Institute of Microbiology and Infection, School of Biosciences, University of Birmingham, Edgbaston, Birmingham, United Kingdom

## Abstract

Pathogen attachment to host cells is a key process during infection, and inhibition of pathogen adhesion is a promising approach to the prevention of infectious disease. We have previously shown that multivalent adhesion molecules (MAMs) are abundant in both pathogenic and commensal bacterial species, mediate early attachment to host cells, and can contribute to virulence. Here, we investigated the efficacy of an engineered bacterium expressing a commensal MAM on its surface in preventing pathogen attachment and pathogen-mediated cytotoxicity in a tissue culture infection model. We were able to dissect the individual contributions of adhesion and interspecific antagonism on the overall outcome of infection for a range of different pathogens by comparison with the results obtained with a fully synthetic adhesion inhibitor. We found that the potential of the engineered bacterium to outcompete the pathogen is not always solely dependent on its ability to hinder host attachment but, depending on the pathogenic species, may also include elements of interspecific antagonism, such as competition for nutrients and its ability to cause a loss of fitness due to production of antimicrobial factors.

## INTRODUCTION

The spread of antibiotic-resistant bacteria is a rising problem and one likely to persist due to the intrinsic mechanism of action of antibiotics generating selective pressure on bacterial populations. As a potential way of addressing this challenge, much work has focused on alternative ways to prevent and treat bacterial infections, including antivirulence approaches (1). These encompass strategies which target the bacterium's ability to colonize and cause disease in a host without interfering with growth or viability (2). Since initial attachment to eukaryotic cells and tissues is an essential early step during colonization of a host, a focus of antivirulence strategies has been the development of adhesion inhibitors (3). A wide range of living (probiotic or engineered), semisynthetic, or fully synthetic adhesion inhibitors has been developed, and ongoing work now focuses on understanding and improving their efficacy (4–6).

Prior work by our group has investigated the use of live and semisynthetic inhibitors based on multivalent adhesion molecules (MAMs), which are an abundant family of bacterial adhesins contributing to initial attachment and virulence in a range of pathogens (7). A related family of mammalian cell entry (MCE) domain-containing proteins is involved in invasion of host cells by mycobacteria and Leptospira spp. (8–10), and peptides targeting this interaction inhibit the uptake of Mycobacterium tuberculosis by host cells (11). Early work focused on materials based on MAM7 from the seafood-borne pathogen Vibrio parahaemolyticus, which demonstrated good efficacy against a broad range of Gram-positive and Gram-negative bacterial infections both *in vitro* and *in vivo* (7, 12–15). Nonadherent Escherichia coli BL21 cells expressing V. parahaemolyticus MAM7 on their surface showed specific adhesion to a range of eukaryotic cells and could prevent infection with V. parahaemolyticus, Vibrio cholerae, Yersinia pseudotuberculosis, or enteropathogenic E. coli (EPEC) (7). Subsequent work showed that this effect could be recapitulated by using a semisynthetic material consisting of recombinant MAM7 chemically coupled to polystyrene beads and that the material efficiently displaced a wider range of clinically relevant pathogens, including Gram-positive bacteria (12, 14). However, V. parahaemolyticus MAM7 was recently shown to interfere with cellular signaling in intestinal epithelial cells and to increase the permeability of intestinal cell junctions *in vitro*, on the basis of its ability to bind to and cluster the signaling lipid phosphatidic acid in host cells (16, 17). Thus, we set out to characterize a putative MAM protein from a well-characterized intestinal commensal, Escherichia coli strain HS, regarding its ability to mediate attachment and displace pathogens from host cells during infection. The E. coli MAM is a protein consisting of 879 amino acids and, like V. parahaemolyticus MAM7, comprises seven MCE domains. However, the overall sequence conservation between the two proteins is low (35%), and ongoing experiments investigating the biochemistry of E. coli MAM-receptor interactions suggest that, unlike V. parahaemolyticus MAM7, E. coli MAM does not utilize phosphatidic acids as a host surface receptor (unpublished results) and therefore may not have the same adverse effects on host cellular signaling.

By using both a live, engineered bacterium and a semisynthetic adhesion inhibitor based on the recombinant MAM protein, we were able to dissect the inhibitory effects of the MAM protein on pathogens and attribute them to either competitive adhesion by MAM-based materials or synergistic effects derived from the engineered bacterial strain. This work demonstrates that competitive displacement of pathogens from the host cell surface can be achieved by using multivalent adhesion molecules with different receptor binding specificities and that, depending on the targeted pathogen, synergistic effects may be derived through the use of a live adhesion inhibitor, rather than semisynthetic material. This finding has wider implications not just for the design of future adhesion inhibitors but also for our understanding of polymicrobial interactions with eukaryotic hosts.

## MATERIALS AND METHODS

### Bacterial strains and growth conditions.

The bacterial strains used were Escherichia coli BL21 that expressed MAM from Escherichia coli HS (MAM^HS^) from a plasmid (pBAD-MAM^HS^) or that was transformed with the empty pBAD plasmid (BL21 control), Pseudomonas aeruginosa PAO1, Enterococcus faecalis ATCC 49452, and Staphylococcus aureus Newman. All strains were grown in Luria Bertani (LB) medium at 37°C prior to infection experiments. The growth medium for BL21 carrying the pBAD plasmids contained 50 μg/ml kanamycin and 0.05% arabinose. For strains transformed with pDP151 expressing mCherry, the medium also contained 100 μg/ml ampicillin. The selective plating media used for determination of the numbers of CFU of specific strains following mixed infections were pseudomonas cetrimide agar for P. aeruginosa, Enterococcosel agar for E. faecalis, and mannitol salt agar for S. aureus.

### Maintenance of tissue culture cells.

HeLa cells were routinely maintained in Dulbecco's modified Eagle's medium (DMEM) supplemented with 10% heat-inactivated fetal bovine serum (HIFBS), 5 mM l-glutamine, 50 μg/ml penicillin, and streptomycin in an atmosphere containing 5% CO_2_ at 37°C.

### Construction of MAM^HS^ expression vectors.

For recombinant expression and purification of the MAM protein from E. coli strain HS (MAM^HS^; GenBank accession number ABV06236.1; GI 157066981) in E. coli BL21, the sequence encoding the soluble portion of the protein (residues 45 to 879) was amplified from E. coli HS genomic DNA. The following primers, containing 5′ BamHI and 3′ XhoI restriction sites, were used: 5′-CAGTGGATCCGACAGTTATCAGGACCGG-3′ and 5′-CAGTCTCGAGTTATTTGGGAAGCGCAGTACC-3′. The insert was cloned into the pGEX-4T-3 plasmid. For expression of the full-length MAM^HS^ protein and surface localization, the complete gene was amplified from E. coli HS genomic DNA using the following primers, containing 5′ NcoI and 3′ HindIII restriction sites: 5′-CAGTCCATGGGACACATGAGTCAGGAAACG-3′ and 5′-CAGTAAGCTTTTATTTGGGAAGCGCAGTACC-3′. The insert was cloned into pBAD/Myc-His, with the Amp^r^ cassette being replaced by a Kan^r^ cassette.

### Expression and purification of GST-MAM^HS^ protein.

Ten milliliters of LB medium supplemented with 100 μg/ml ampicillin was inoculated with a colony of E. coli BL21 transformed with pGEX-4T-3 containing the MAM^HS^ construct and incubated with shaking at 37°C overnight. Following overnight incubation, 5 ml of the LB broth culture was added per 500 ml of autoinduction medium supplemented with 100 μg/ml of ampicillin, grown at 225 rpm and 37°C until the culture reached an optical density at 600 nm (OD_600_) of 0.6 to 0.7, and then incubated at 20°C overnight. Following incubation, the cultures were centrifuged at 6,000 rpm for 10 min. The bacterial pellets were resuspended in 25 ml of glutathione *S*-transferase (GST) lysis buffer (25 mM Tris, pH 7.5, 150 mM NaCl, 1 mM EDTA, protease inhibitors, lysozyme, DNase I), 20 mM sodium cholate was added, and then the suspension was incubated at 4°C overnight. The suspension was sonicated on ice twice for 60 s each time (5 s on and 5 s off; 60% output) and centrifuged at 10,000 rpm for 30 min at 4°C. The pellets were discarded, and the supernatants were filtered through a 0.45-μm-pore-size filter. Two milliliters of washed glutathione beads was added to the supernatant, and the mixture was rotated at 4°C for 2 h. After the incubation, 20 ml of binding buffer (25 mM Tris, pH 7.5, 150 mM NaCl, 1 mM EDTA) was added to the column containing the beads, and this wash step was repeated twice. Elution buffer (1.5 ml; 10 mM reduced glutathione, 50 mM Tris-HCl, pH 8.0) was added to the beads, the mixture was incubated for 15 min, and then the elution buffer was drained; this step was repeated twice. Finally, 1.5 ml of elution buffer containing 100 mM reduced glutathione was added to the beads, the mixture was incubated for 15 min, and then the elution buffer was drained. Glutathione was removed from the GST-fusion proteins by dialysis at 4°C overnight. Protein purity was ∼90%, as determined by SDS-PAGE.

### Coupling of recombinant GST-MAM^HS^ protein to polymer beads.

Fluorescent blue beads of 2 μm in diameter (Sigma) were coupled to the GST-MAM^HS^ protein or the GST protein (control). The detailed protocol for the coupling procedure has been described elsewhere (18). Briefly, the washed beads were resuspended in phosphate-buffered saline (PBS) and incubated with 10 mM sulfo-succinimidyl 4-(*p*-maleimidophenyl)butyrate for 1 h at 25°C while they were rotated. After the activated beads were washed, they were resuspended in PBS containing a final concentration of 6 μM protein and incubated for 2 h at 25°C. Cysteine (50 mM) was added to quench the reaction, and the beads were washed twice with PBS and resuspended in fresh PBS to give the final product. The final protein concentration of the protein-coupled beads used in the experiments was 500 nM, and 2 × 10^11^ beads/ml were used.

### Attachment assays.

To test the ability of E. coli BL21 to adhere to host cells, HeLa cells were seeded at a concentration of 150,000 cells/ml a day prior to the experiment, and E. coli BL21 cells transformed with pBAD/Myc-His-MAM^HS^ (BL21+MAM^HS^) or empty pBAD (control) were grown in LB broth containing kanamycin on the day before the experiment. For the attachment experiment, E. coli BL21 was grown in LB broth containing kanamycin, and when the OD_600_ reached 0.6, protein expression was induced by adding arabinose to 0.05% and incubating at 37°C for a further 4 h. HeLa cells were infected with E. coli BL21 as a control or E. coli BL21+MAM^HS^ at a multiplicity of infection (MOI) of 100 for 1 h. Following the infection, the DMEM was removed and the HeLa cells were washed three times with PBS to remove unattached bacteria. Triton X-100 (0.5%) in PBS was added to lyse the HeLa cells, and then serial dilutions were prepared, cultured on LB agar, and incubated at 37°C for 20 h, followed by counting of the number of colonies.

### Competition experiments.

On the day before the experiment, HeLa cells were seeded at a concentration of 150,000 cells/ml into 24-well plates. Wells for untreated HeLa cells (negative control) and for Triton X-100 lysis (positive control) were also prepared. The bacteria were grown in LB broth overnight. When the E. coli BL21 strains were used to compete with pathogenic bacteria, on the day of the experiment, the strains were grown and protein expression was induced as described above. Tissue culture cells were washed with PBS prior to the addition of E. coli BL21 at an MOI of 100 and either S. aureus, E. faecalis, or P. aeruginosa at an MOI of 10, and the cells were incubated at 37°C for 4 h. When beads were used in competition experiments, HeLa cells were infected by adding the pathogens at an MOI of 10 together with 2 × 10^11^ beads/ml (GST or GST-MAM^HS^) containing protein at a final concentration of 500 nM. Following incubation for 4 h, supernatant samples were removed for cytotoxicity measurements, and cells were treated as described below for either counting of attached bacteria or microscopy.

### Cytotoxicity measurements.

The lactate dehydrogenase (LDH) released into the medium was quantified using an LDH cytotoxicity detection kit (TaKaRa), as described in the kit protocol. The data were expressed as the percent cytotoxicity normalized to that for untreated cells (0% lysis) and Triton X-100-lysed cells (100% lysis) and was calculated by the following formula: percent cytotoxicity = 100 × [(OD_490_ for experimental release − OD_490_ for spontaneous release)/(OD_490_ for maximum release − OD_490_ for spontaneous release)].

### Quantification of attached bacteria.

Medium was removed and HeLa cells were washed three times with PBS. One milliliter of 0.5% Triton X-100 in PBS was added to each well to lyse the HeLa cells. Serial dilutions of lysed samples were cultured on selective medium as described above, and the colonies were counted after incubation at 37°C for 20 h.

### Fluorescence microscopy.

Following removal of the supernatants, HeLa cells were washed three times with sterile PBS and fixed using 3.2% formaldehyde in PBS for 15 min at room temperature. After fixation, the cells were washed with PBS and treated with 0.1% Triton X-100 in PBS for 5 min to permeabilize the cells. The cells were then stained with either rhodamine-phalloidin or Alexa Fluor 488-phalloidin and Hoechst for 10 min in the dark. Samples were mounted using ProLong Gold antifade mounting medium and imaged using a Nikon Ti Eclipse microscope. Images were processed using ImageJ and Corel Draw X5 software.

## RESULTS

### Characterization of an engineered bacterium and a synthetic adhesion inhibitor based on an MAM from commensal E. coli isolate HS.

As an extension of previous work on multivalent adhesion molecules (MAMs) derived from pathogenic Gram-negative bacterial species (7, 14), we set out to characterize a MAM derived from a commensal E. coli isolate and to characterize its potential for and mode of pathogen displacement from host cells in more detail. For this purpose, we constructed two types of materials. First, we constructed a conditionally adhesive strain of E. coli, further referred to as BL21+MAM^HS^. This strain was made by transforming E. coli BL21 with a plasmid expressing the MAM from commensal E. coli isolate HS from an arabinose-inducible promoter, thus enabling its surface display in BL21 cells. E. coli BL21 transformed with pBAD alone served as a negative control. BL21+MAM^HS^ was designed to investigate pathogen displacement from host cells and to capture interactions based on attachment, as well as any additional effects based on intraspecific interactions between BL21 and pathogenic bacteria. Second, we constructed a synthetic material, consisting of polymer beads chemically coupled to recombinant GST-tagged MAM derived from E. coli HS (MAM^HS^ beads). This was made to capture interactions between MAM and pathogens solely based on attachment competition and to distinguish these from secondary effects due to intraspecific bacterial interactions. In this case, beads coupled to the GST tag alone (control beads) served as a negative control.

**FIG 1:**
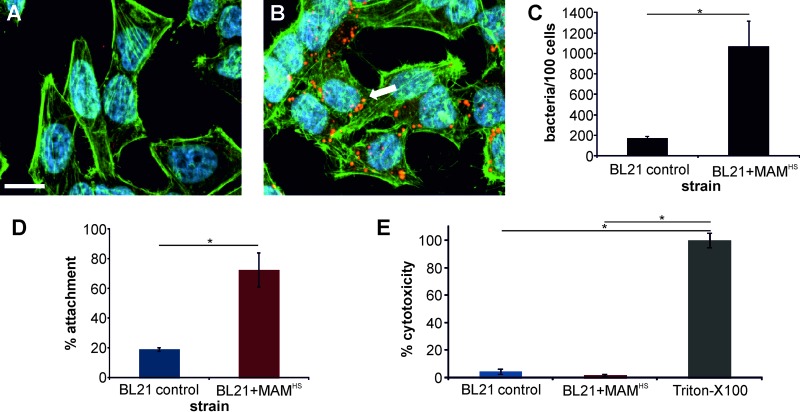
MAM^HS^ mediates adhesion of nonadherent E. coli cells to host cells and is nontoxic. (A and B) Attachment of E. coli BL21 cells expressing mCherry (red) and transformed with pBAD (A) or pBAD containing MAM from E. coli HS (BL21+MAM^HS^) (B) to HeLa cells. Cells were fixed and stained for actin (green) and DNA (blue) following a 1-h infection. The arrow indicates attached bacteria. Bar, 20 μm. (C) The numbers of attached bacteria were quantified by image analysis, and the data represent the means ± SEMs based on analysis of at least 300 HeLa cells over three independent experiments. Significance was determined using Student's *t* test. *, *P* ≤ 0.05. (D) The attachment of BL21 cells to HeLa cells was also quantified by dilution plating, and the results are expressed in relation to the CFU counts from the input controls (100%). (E) The cytotoxicity of BL21 toward HeLa cells was determined using LDH release assays and is normalized to that toward untreated (0%) and Triton X-100-treated (100%) controls. The results in panels D and E are means ± SEMs (*n* = 3), and significance was determined using Student's two-tailed *t* test. *, *P* ≤ 0.05.

**FIG 2:**
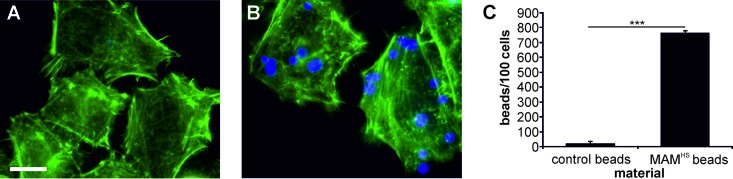
Polymer beads coupled to MAM^HS^ adhere to host cells. (A and B) Attachment of polystyrene beads (blue) coupled to GST (A) or GST-MAM^HS^ (B) to HeLa cells (F-actin stained green). Bar, 10 μm. (C) The numbers of attached bacteria were quantified by image analysis, and data represent the means ± SEMs based on analysis of at least 300 HeLa cells over three independent experiments. Significance was determined using Student's *t* test. ***, *P* ≤ 0.001.

Initially, we characterized the adhesive properties of BL21+MAM^HS^ and BL21 control cells toward host cells using fluorescence imaging (Fig. 1A to C) as well as enumeration of attached bacteria by dilution plating (Fig. 1D). To visualize the attached bacteria, both BL21 control and BL21+MAM^HS^ cells were cotransformed with a plasmid expressing mCherry. The surface expression of MAM^HS^ conferred adhesive properties to BL21, with an average of 1,070 ± 250 bacteria being attached per 100 host cells, while BL21 bacteria lacking MAM^HS^ showed little attachment (average, 170 ± 20 bacteria per 100 host cells). Enumeration of attached bacteria by dilution plating revealed a similar picture: 19% of the BL21 control bacteria and 73% of the BL21+MAM^HS^ bacteria attached relative to the input amount of bacteria. Neither the BL21 control nor the BL21+MAM^HS^ strain displayed any cytotoxicity toward host cells (Fig. 1E). MAM^HS^ beads were also adhesive toward host cells (average, 767 beads/100 cells bound), whereas control beads were not (average, 20 beads/100 cells attached) (Fig. 2).

Since both BL21+MAM^HS^ and MAM^HS^ beads were able to adhere to host cells, we next tested their ability to displace three different Gram-positive and Gram-negative bacterial pathogens from the host cell surface. Pseudomonas aeruginosa, Enterococcus faecalis, and Staphylococcus aureus are all causative agents of wound infections and surgical site infections (19–21), and infections caused by these bacteria are increasingly difficult to treat due to intrinsic and acquired antimicrobial resistance (22, 23). Since we are interested in exploiting MAM mimetics to inhibit infections, we characterized the interactions of BL21+MAM^HS^ and MAM^HS^ beads with these species and with epithelial cells in detail, to test the efficacy and mode of action of both types of inhibitors and evaluate which approach may be more beneficial.

### The inhibitory effect of BL21+MAM^HS^ on Pseudomonas aeruginosa is solely based on competitive replacement from surface receptors.

**FIG 3:**
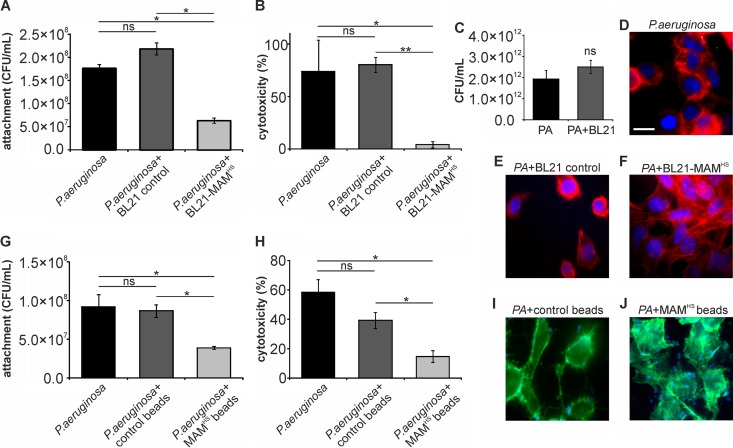
The inhibitory effect of BL21+MAM^HS^ on Pseudomonas aeruginosa (PA) is based on competitive replacement from surface receptors. HeLa cells were infected with P. aeruginosa (MOI, 10) alone or in the presence of either the BL21 control or the BL21+MAM^HS^ strain. Alternatively, HeLa cells were infected with P. aeruginosa (MOI, 10) alone or in the presence of either GST-coupled control beads or MAM^HS^-coupled beads. After 4 h, the numbers of attached P. aeruginosa cells were determined by selective plating (A, G) and cytotoxicity toward HeLa cells was determined by LDH release assays (B, H). Cytotoxicity was normalized against that for uninfected (0%) and Triton X-100-lysed (100%) samples. The morphology of infected HeLa cells following infections was determined by imaging. The staining in panels D to F shows DNA (blue) and F-actin (red). Bar, 20 μm. The staining in panels I and J shows beads (blue) and F-actin (green). (C) Mixed planktonic cultures of P. aeruginosa and BL21 were grown for 4 h, and the number of P. aeruginosa cells was determined by selective plating. Results are means ± SEMs (*n* = 3), and significance was determined using Student's two-tailed *t* test on raw data. *, *P* ≤ 0.05; **, *P* ≤ 0.01; ns, no significant difference.

**FIG 4:**
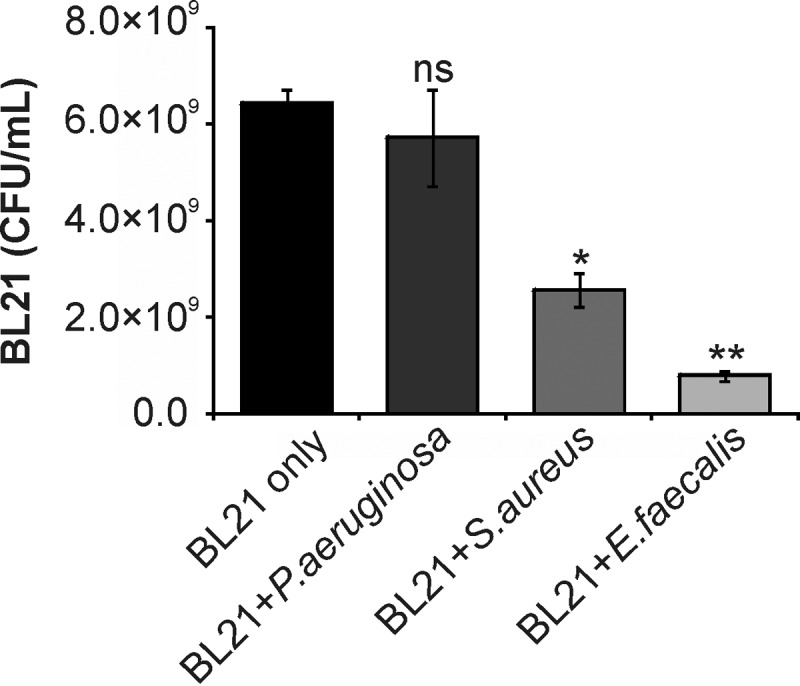
Interactions between pathogens and E. coli BL21 during growth. E. coli BL21 was grown alone or as mixed cultures of E. coli BL21 and either E. faecalis, P. aeruginosa, or S. aureus for 4 h. The numbers of E. coli BL21 cells were determined by selective plating. Results are means ± SEMs (*n* = 3), and significance was determined using Student's two-tailed *t* test on raw data. *, *P* ≤ 0.05 compared to the growth of BL21 alone; **, *P* ≤ 0.01 compared to the growth of BL21 alone; ns, no significant difference compared to the growth of BL21 alone.

HeLa epithelial cells were incubated with P. aeruginosa at a multiplicity of infection (MOI) of 10 or with both P. aeruginosa (MOI, 10) and the BL21 control or BL21+MAM^HS^ (MOI, 100). Following a 4-h infection, the attachment of P. aeruginosa and the resulting cytotoxicity on host cells were quantified. The presence of BL21+MAM^HS^ led to a significant decrease in both the number of attached P. aeruginosa cells (3-fold reduction) and the resulting cytotoxicity (18-fold reduction) for host cells, while no significant changes were observed with P. aeruginosa in the presence of the BL21 control (Fig. 3). We further studied the intraspecific interactions between E. coli BL21 and P. aeruginosa. Coincubation of both species in liquid broth had no effect on the numbers of either P. aeruginosa (Fig. 3C) or BL21 (Fig. 4) cells. Infection of epithelial cells with P. aeruginosa in the presence of MAM^HS^ beads but not control beads equally led to a significant decrease in both the level of P. aeruginosa attachment to host cells (2-fold reduction) and cytotoxic effects on host cells (4-fold reduction) (Fig. 3G and H). These results were also confirmed by visual inspection of infected cells (Fig. 3D to F, I, and J).

### The inhibitory effect of BL21+MAM^HS^ on Enterococcus faecalis is due to a combination of attachment competition and competition for nutrients.

**FIG 5:**
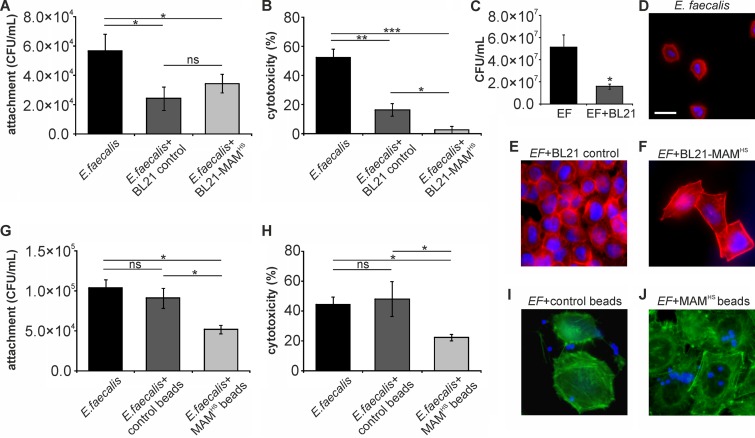
The inhibitory effect of BL21+MAM^HS^ on Enterococcus faecalis (EF) is due to a combination of attachment competition and competition for nutrients. HeLa cells were infected with E. faecalis (MOI, 10) alone or in the presence of either the BL21 control or the BL21+MAM^HS^ strain. Alternatively, HeLa cells were infected with E. faecalis (MOI, 10) alone or in the presence of either GST control beads or MAM^HS^-coupled beads. After 4 h, the numbers of attached E. faecalis cells were determined by selective plating (A, G) and cytotoxicity toward HeLa cells was determined by LDH release assays (B, H). Cytotoxicity was normalized against that for uninfected (0%) and Triton X-100-lysed (100%) samples. The morphology of infected HeLa cells following infections was determined by imaging. The staining in panels D to F shows DNA (blue) and F-actin (red). Bar, 20 μm. The staining in panels I and J shows the beads (blue) and F-actin (green). (C) Mixed planktonic cultures of E. faecalis and BL21 were grown for 4 h, and the number of E. faecalis cells was determined by selective plating. Results are means ± SEMs (*n* = 3), and significance was determined using Student's two-tailed *t* test on raw data. *, *P* ≤ 0.05; **, *P* ≤ 0.01; ***, *P* ≤ 0.001; ns, no significant difference.

A previous study by our group (14) showed that, due to overlap in the host receptors utilized by MAMs and many Gram-positive bacterial fibronectin-binding adhesins, MAMs also have the ability to displace Gram-positive bacterial pathogens from the host cell surface. We therefore tested the effect of BL21+MAM^HS^ on infection with E. faecalis. Both BL21+MAM^HS^ and the BL21 control strain lacking MAM^HS^ significantly decreased the number of host cell-bound E. faecalis cells (2-fold reduction; Fig. 5A). They both also decreased E. faecalis-mediated cytotoxicity toward host cells, but the effect of BL21+MAM^HS^ was greater (20-fold reduction) than that observed with the BL21 control strain (3-fold reduction) (Fig. 5B). Visualization of the infection further confirmed these results (Fig. 5D to F). Quantification of both E. coli and E. faecalis following a 4-h coincubation in liquid broth showed that both E. coli and E. faecalis were significantly inhibited by each other's presence (Fig. 4 and 5C). To distinguish these intraspecific bacterial interactions from inhibitory effects due to attachment competition, we investigated the effect of MAM^HS^ beads on E. faecalis infection. While MAM^HS^ beads decreased the attachment of E. faecalis cells to host cells (2-fold reduction) as well as cytotoxicity (2-fold reduction), control beads had no effect on either readout (Fig. 5G and H). Visualization of bead-based inhibition confirmed these results; attachment of MAM^HS^ beads to host cells had a protective effect, and more epithelial cells than cells infected in the presence of control beads maintained an intact morphology (Fig. 5I and J).

### The inhibitory effect of BL21+MAM^HS^ on Staphylococcus aureus is due to a combination of attachment competition and interspecific antagonism.

**FIG 6:**
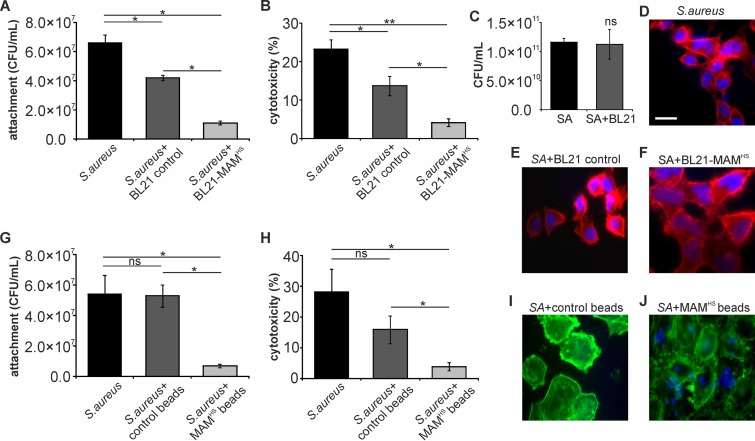
The inhibitory effect of BL21+MAM^HS^ on Staphylococcus aureus (SA) is due to a combination of attachment competition and interspecies antagonism. HeLa cells were infected with S. aureus (MOI, 10) alone or in the presence of either the BL21 control or the BL21+MAM^HS^ strain. Alternatively, HeLa cells were infected with S. aureus (MOI, 10) alone or in the presence of either GST-coupled control beads or MAM^HS^-coupled beads. After 4 h, the numbers of attached S. aureus cells were determined by selective plating (A, G) and cytotoxicity toward HeLa cells was determined by LDH release assays (B, H). Cytotoxicity was normalized against that for uninfected (0%) and Triton X-100-lysed (100%) samples. The morphology of infected HeLa cells following infections was determined by imaging. The staining in panels D to F shows DNA (blue) and F-actin (red). Bar, 20 μm. The staining in panels I and J shows the beads (blue) and F-actin (green). (C) Mixed planktonic cultures of S. aureus and BL21 were grown for 4 h, and the number of S. aureus cells was determined by selective plating. Results are means ± SEMs (*n* = 3), and significance was determined using Student's two-tailed *t* test on raw data. *, *P* ≤ 0.05; **, *P* ≤ 0.01; ns, no significant difference.

S. aureus is a Gram-positive opportunistic pathogen which has previously been shown to be inhibited by the binding of bead-coupled V. parahaemolyticus MAM to host cells. A similar decrease of S. aureus adhesion to host cells (8-fold reduction) and cytotoxicity toward host cells (7-fold reduction) was observed here when we used bead-coupled MAM^HS^ as the competing moiety. In contrast, incubation in the presence of control beads had no significant effect on either S. aureus attachment or cytotoxicity toward host cells (less than 2-fold reduction; Fig. 6G to J). Interestingly, when we used BL21+MAM^HS^ as the inhibitor against S. aureus, a significant effect on attachment and cytotoxicity was observed even with the control strain (Fig. 6A to F). Coincubation with BL21 lacking MAM^HS^ led to a slight decrease in both attachment and cytotoxicity (∼2-fold). However, the inhibitory effect was more pronounced with BL21+MAM^HS^ (6-fold reduction of both adherence and cytotoxicity), and the effects achieved with the BL21 control and BL21+MAM^HS^ strains were significantly different both visually and on the basis of the results of quantitative assays (Fig. 6A, B, and D to F). When S. aureus and E. coli BL21 were grown together in the absence of host cells, S. aureus growth was unaffected (Fig. 6C), but the growth of BL21 was significantly diminished (Fig. 4). We performed follow-up experiments to identify the nature of the inhibition that S. aureus exerts on E. coli. Filtered supernatant isolated from S. aureus cultures was spotted on filter disks and caused a visible zone of inhibition on a lawn of E. coli after 16 h of incubation at 37°C (data not shown). Thus, S. aureus likely produces a secreted factor which inhibits the growth of E. coli BL21.

## DISCUSSION

The ability of bacteria to intimately adhere to host cells and tissues is a conserved feature in bacteria capable of a host-associated life style and essential for successful interactions between bacteria and the host. This makes adherence a potential target for antivirulence approaches, and we have previously shown that materials based on the V. parahaemolyticus protein MAM7 can efficiently displace a broad range of Gram-positive and Gram-negative bacterial pathogens of clinical importance. Due to the potential interference of the initially used adhesin, MAM7 from V. parahaemolyticus, with host cell function and its ability to enhance transcellular permeability in cultured intestinal epithelial cells, we shifted our focus to an MAM protein from the well-characterized commensal HS strain of E. coli.

Commensal strains of E. coli can strongly adhere to epithelial cells without any ill effects, and this property has been shown to be mediated by a range of adhesins, including fimbriae and flagella (24, 25). These adhesive properties have previously been shown to be essential for commensal strains' ability to displace enteric pathogens, such as Salmonella enterica serovar Typhimurium and pathogenic E. coli (25–27). By heterologous surface expression of MAM from E. coli HS in the surrogate, nonadherent strain E. coli BL21, we created an engineered, adhesive strain for which adherence is solely based on MAM. We also created a semisynthetic material consisting of polystyrene beads with dimensions similar to those of E. coli chemically and directionally coupled to MAM from E. coli HS (MAM^HS^). This further allowed us to dissect any observed interactions between the materials and pathogens and attribute them to either adhesion or additional intraspecies interactions between E. coli and the pathogens used.

The results obtained with both BL21+MAM^HS^ and the bead-based inhibitor showed that MAM^HS^ mediates specific and strong adherence to host cells. The binding affinity and avidity of MAM^HS^ seem to be comparable to those of V. parahaemolyticus MAM7 and the MAM protein from enteropathogenic E. coli (EPEC), as it mediates the adherence of otherwise nonadherent BL21 to host cells to a similar extent as MAM7 and EPEC MAM (7).

The ability to displace pathogens from the host surface appears to be a conserved feature among MAMs, as BL21+MAM^HS^ was able to decrease both the attachment and cytotoxicity of all three pathogens tested here. As is the case with Vibrio MAM7-based inhibitors (13, 14), MAM^HS^ was able to displace both Gram-positive and Gram-negative bacterial pathogens. The biochemical basis of this behavior will require further investigation, as our initial characterization of MAM^HS^ host receptor binding has demonstrated that it does not have the same ligand binding specificity as Vibrio MAM7, which utilizes phosphatidic acids as a main receptor (28). While the lack of MAM^HS^ affinity toward phosphatidic acids means that the protein likely does not interfere with host cell signaling in the same way that V. parahaemolyticus MAM7 does, further work is needed to identify its host surface receptor.

Previous work comparing the inhibitory properties of live bacteria expressing MAM7 and bead-coupled MAM7 have largely yielded similar results with both materials. Both materials showed broad efficacy against V. parahaemolyticus, V. cholerae, Yersinia pseudotuberculosis, enteropathogenic E. coli, Pseudomonas aeruginosa, Klebsiella pneumoniae, and Acinetobacter spp. but had comparable activities against these bacteria, and no synergies were observed with the use of live bacteria over bead-based materials (7, 13). These results were recapitulated here with P. aeruginosa, in that both bead-based and live materials had comparable effects on this species, and no effects were observed with the BL21 control strain (12). In the present study, synergies due to intraspecific interactions were restricted to those against the Gram-positive bacterial isolates, and none of these were previously investigated with live adhesion inhibitors. However, the effects observed here with the bead-based adhesion inhibitor on S. aureus strain Newman were similar to the effects that we previously observed with MAM7 beads and S. aureus USA300 (14).

A comparison of the effects of the bacterium- and bead-based inhibitors on infection of host cells with E. faecalis showed a clear synergistic effect of the engineered bacteria. While the bead-based inhibitor halved the level of E. faecalis-mediated cytotoxicity, BL21+MAM^HS^ decreased the toxicity approximately 10-fold (Fig. 5). Further investigation showed that E. faecalis and E. coli limit each other's growth, most likely through competition for shared nutrients (Fig. 4 and 5C). While this implies a similar limitation of the growth of E. coli during the competition experiment, this growth limitation does not seem to affect its potential to outcompete E. faecalis, most likely due to the initial excess of E. coli over E. faecalis in the experiment.

In the case of S. aureus, the bacterium- and bead-based inhibitors had comparable overall effects on the outcome of infection (a 5-fold versus 4- to 5-fold reduction in S. aureus-mediated cytotoxicity, respectively). However, coincubation with the BL21 control also led to a partial decrease in both adherence and cytotoxicity. The interaction between S. aureus and E. coli seems to be characterized by a one-sided inhibition of E. coli growth by S. aureus, whereas E. coli has no inhibitory effect on S. aureus growth (Fig. 4 and 6C). The basis of this one-sided inhibition could be production of a bacteriocin or other secreted inhibitory factors, such as bacteriolytic enzymes, by S. aureus, since the sterile supernatant of an S. aureus culture is sufficient to cause a zone of clearance on a lawn of E. coli BL21 cells. S. aureus is known to produce a wide range of antimicrobial factors, including several which show activity against Gram-negative bacteria (29, 30). While this production of antimicrobials by S. aureus may lead to a decrease in its capacity to adhere and cause cytotoxicity, this effect does not ultimately lead to synergy with MAM-mediated competitive displacement, since the number of S. aureus cells does not decrease through the presence of BL21. The use of a bacteriocin-resistant strain of E. coli as a surrogate may be a way to maintain the number of E. coli cells during the competition and may thus encourage synergistic effects.

In summary, this study demonstrates that competitive replacement of pathogens is a shared feature of MAMs and that the use of engineered live, adhesive bacterial strains may have beneficial effects on the outcome of the competition, beyond the competitive binding of MAM to host cells. These synergies depend on the nature of the competing pathogen and may involve competition for nutrients or trade-offs between the production of antimicrobials and the production of colonization and virulence factors. These added layers of complexity are an important consideration not only in the future design of adhesion inhibitors but also in thinking about polymicrobial interactions in the context of a host in general.
